# Whole-Community Facilitation Regulates Biodiversity on Patagonian Rocky Shores

**DOI:** 10.1371/journal.pone.0024502

**Published:** 2011-10-13

**Authors:** Brian R. Silliman, Mark D. Bertness, Andrew H. Altieri, John N. Griffin, M. Cielo Bazterrica, Fernando J. Hidalgo, Caitlin M. Crain, Maria V. Reyna

**Affiliations:** 1 Department of Biology, University of Florida, Gainesville, Florida, United States of America; 2 Department of Ecology and Evolutionary Biology, Brown University, Providence, Rhode Island, United States of America; 3 Department of Biology, University of Patagonia, Puerto Madryn, Chibut, Argentina; 4 Departament of Biology, National University of Mar del Plata, Mar del Plata, Argentina; Northeastern University, United States of America

## Abstract

**Background:**

Understanding the factors that generate and maintain biodiversity is a central goal in ecology. While positive species interactions (i.e., facilitation) have historically been underemphasized in ecological research, they are increasingly recognized as playing important roles in the evolution and maintenance of biodiversity. Dominant habitat-forming species (foundation species) buffer environmental conditions and can therefore facilitate myriad associated species. Theory predicts that facilitation will be the dominant community-structuring force under harsh environmental conditions, where organisms depend on shelter for survival and predation is diminished. Wind-swept, arid Patagonian rocky shores are one of the most desiccating intertidal rocky shores ever studied, providing an opportunity to test this theory and elucidate the context-dependency of facilitation.

**Methodology/Principal Findings:**

Surveys across 2100 km of southern Argentinean coastline and experimental manipulations both supported theoretical predictions, with 43 out of 46 species in the animal assemblage obligated to living within the matrices of mussels for protection from potentially lethal desiccation stress and predators having no detectable impact on diversity.

**Conclusions/Significance:**

These results provide the first experimental support of long-standing theoretical predictions and reveal that in extreme climates, maintenance of whole-community diversity can be maintained by positive interactions that ameliorate physical stress. These findings have important conservation implications and emphasize that preserving foundation species should be a priority in remediating the biodiversity consequences of global climate change.

## Introduction

Biodiversity and the critical services it provides are under global siege from human impacts [1]–[4]. Climate change, habitat destruction, over-harvesting, pollution, and species introductions are leading anthropogenic forces driving declines in species populations, diversity and ecosystem services [1], [5]–[8]. Recognition of these growing threats to biodiversity has sparked added attention to both elucidating the key biological and physical factors that structure local species richness and evenness [9]–[10], and the context-dependency of their relative impacts. Refined understanding of these issues will be critical for predicting not only how biodiversity will be impacted by an increasingly variable and changing environment but also the potentially compounding effects of losing key diversity-regulating species interactions [11]–[12].

While negative species interactions (e.g., competition, predation) have long been recognized as important controls of local biodiversity [13]–[15], the role of positive interactions (mutualisms, commensalisms; i.e., facilitation) has historically received far less attention [16]. Dominant habitat-forming organisms (foundation species *sensu* [17]; e.g., oysters, corals, trees and grasses) are perhaps the most conspicuous examples of species that play critical roles in structuring ecological communities via positive interactions. Through the formation of physical structure and often complex interstitial spaces, foundation species buffer other species against biotic and abiotic stress and produce an array of micro-habitats that can facilitate persistence of associated organisms, and therefore promote increased biodiversity in the communities they dominate [17]–[19]. The importance of foundation species for community structure varies among systems and contexts, but can be conceptualized as functions of the proportion of species in the community that are facilitated (breadth) by the foundation species and the strength of those positive interactions. At one end of this continuum, a small proportion of species may derive weak, facultative benefits from a foundation species, and at the other end, whole-community facilitation occurs and co-existing species are obligately dependent on a foundation species [20]. Theory (i.e., the environmental stress model) suggests that the importance of foundation species (breadth and strength of facilitation) increases with environmental stress [16], [21], [22] as more species become increasingly dependent on buffered conditions for survival. Under highly stressful environmental conditions, foundation species are thus expected to play a critical role in structuring communities and maintaining biodiversity.

While foundation species facilitate associated species, their dominance of primary resources (e.g., space) can also result in the competitive exclusion of species with overlapping resource requirements [13], [23]. By suppressing competitively dominant foundation species, ‘keystone’ predators have been shown to facilitate co-existence of competitors in a variety of ecosystems (e.g., [13], [24]). The importance of keystone predation in structuring communities is expected to shift with the level of environmental stress; in contrast to facilitation, it is predicted to diminish in strength and importance with increasing environmental stress as predators become less abundant and effective [21] under high levels of physiological stress. To our knowledge however, no studies have concomitantly examined the importance of diversity-facilitation by foundation species and the effects of predators under high levels of physical stress. If indeed the importance of foundation species buffering ramps up at these higher levels of stress while keystone predation slows or shuts down, then conservation managers would be compelled to consider allocating increased effort into conserving and restoring foundation species in a world where human-induced global changes are rapidly ratcheting-up environmental stressors.

We performed the current study on the rocky shores of Argentinean Patagonia (41–55° S, 63–70° W). These shores are subjected to dry, persistent winds of “The Roaring 40 s”, which flow relatively unimpeded by landmasses around much of the Southern Hemisphere. These intense winds bring unrivaled climatic conditions to Argentinean Patagonia shorelines with daily average wind speeds >30 km/h (commonly >60 km/h), annual rainfall <18 cm/yr, and humidity typically <40% [25]. Combined, these atmospheric forces generate a desiccation stress higher than that measured on any other previously studied rocky shore system [25]. In light of the predictions of environmental stress models (see above), we hypothesized that facilitation by foundation species rather than predation would play the most critical role in controlling biodiversity on the exposed rocky shores of Patagonia where the intertidal communities are subjected to a diverse regime of stressful climatic conditions.

**Figure 1:**
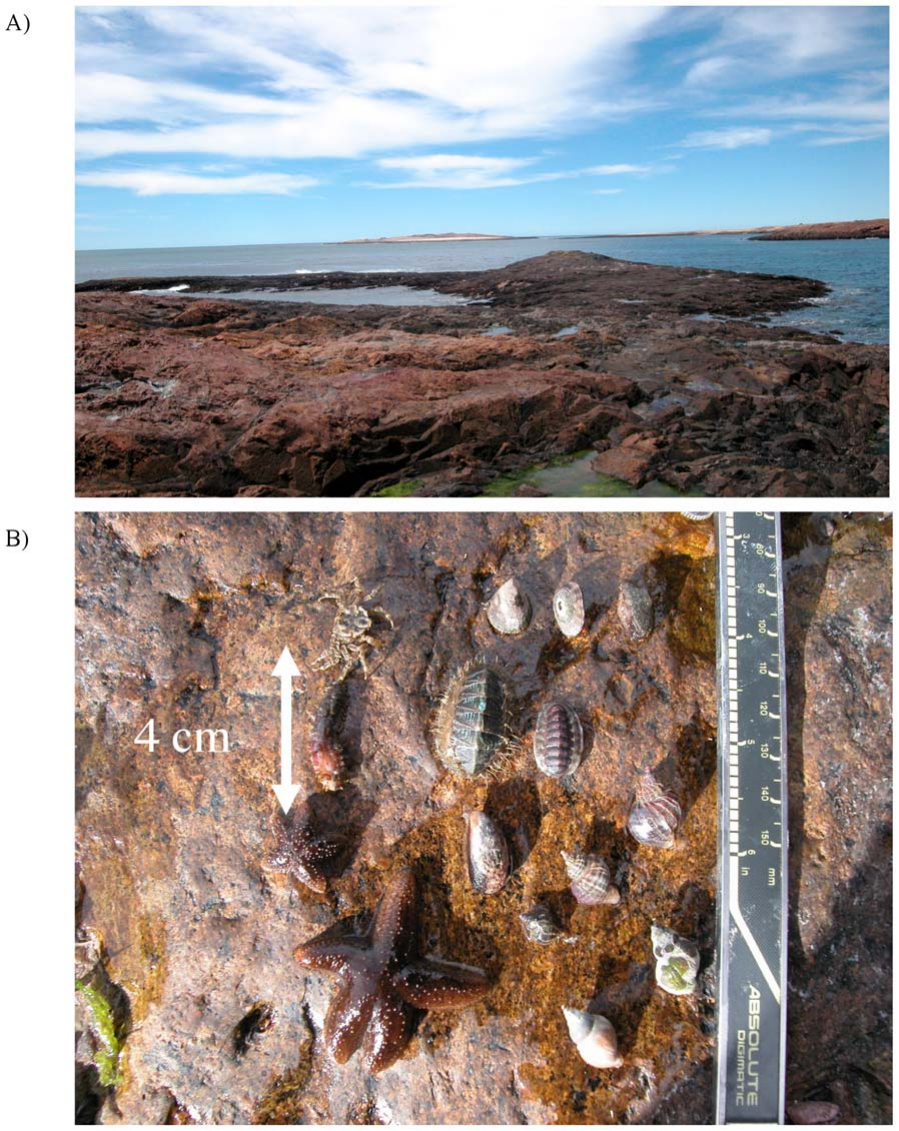
Images of habitat created by mussels, and the animals sheltered within. (A) Seacape view of mussel-dominated, Patagonian exposed rocky shores. (B) Typical diminutive, invertebrate species living only within the mussel matrix.

Initial observations and experiments at the Natural Protected Area of Cabo Dos Bahias (44°44′S, 65°40′W), revealed a diverse community of >40 animal species, including mussels, amphipods, isopods, anemones, chitons, snails, crabs, limpets, polycheates, nudibranchs, brittle stars, nemerteans, barnacles, and seastars (Fig. 1). All but one of these organisms (an invasive barnacle) were found exclusively within or nestled tightly on top of (2 limpets) a biogenic matrix created by the mussel (*Perumytilus purpuratus*). Within this biomatrix, there was ample intra-mussel space for the shelter and movement of symbionts, as it was between 2–4 shell layers thick, predominantly sediment-free, and covered nearly the entire intertidal zone (>95%) of exposed headlands, from the low to high tide marks (Fig. 1; [25]). In addition, we found no evidence for the presence of abundant predators (e.g., large seastars, crabs and drilling snails) on the rock and mussel bed surfaces that are so common on other exposed rocky shores throughout the world [26]–[29]. Given these initial observations, and recent work showing the prominence of whole-community facilitation under high environmental stress [20], [30], we hypothesized that this intertidal mussel could be acting as an obligate foundation species on wind-swept Patagonian shorelines by providing community-wide refugia from potentially lethal climatic stressors.

To begin to test our hypothesis that facilitation by mussel beds (via desiccation stress amelioration) regulates local biodiversity on Argentinean Patagonia rocky shores, we initially used an observational, comparative approach and sampled species diversity in the mussel matrix and adjacent bare areas in high and low intertidal habitats at two wave-exposed headlands in Cabo Dos Bahias and 8 other exposed rocky coast sites along the Patagonian coast, spanning >2100 km. To experimentally test our hypothesis, we conducted both field survivorship (with 7 species) and disturbance-recovery (with the entire community) experiments both with and without mussel beds and consumers at replicated exposed rocky shore sites in Cabo Dos Bahias from 2003–2005.

To test how predators (seastars) within the mussel matrix impact community development, we performed a 1.5 yr seastar removal experiment at both Cabo Dos Bahias sites in the low intertidal zone where seastar abundance is highest. Finally to test how mussels mitigate desiccation stress on Patagonian shores and to parse out the relative importance of sun vs. wind block by mussels in reducing physical stress, we performed a sponge-evaporation study at the interface between the mid and low intertidal zones at two exposed headland sites in the Cabo Dos Bahias preserve.

## Materials and Methods

### Site Description

We obtained permits for our work on Argentinean shorelines from the Argentinean Department of National Parks. For a copy of the permit (no permit number was issued), please contact F. Hidalgo (fernandohidalgo2003@yahoo.com.ar). The primary site of this study was in the Natural Protected Area of “Cabo Dos Bahias” (44°44′S, 65°40′W), on the north end of the Gulf of San Jorge, Patagonia, Argentina. This protected area is a land-to-sea reserve situated along the Patagonian steppe and characterized by an arid and desert-like climate with low precipitation (∼18 cm/yr), mean temperatures of 12.5 C° (maximum of 39 C° and minimum of −7.5 C°), and strong, dry, southwest winds, with mean velocities of 25–35 km/h and maximums routinely >60 km/h. Desiccation stress is accordingly severe and among the highest recorded for rocky shore communities [25]. Tides are semi-diurnal, with average amplitude of 3.4 m, and wave stress on exposed headlands is comparable to that experienced on rocky shores on the west coast of the U.S.

**Figure 2:**
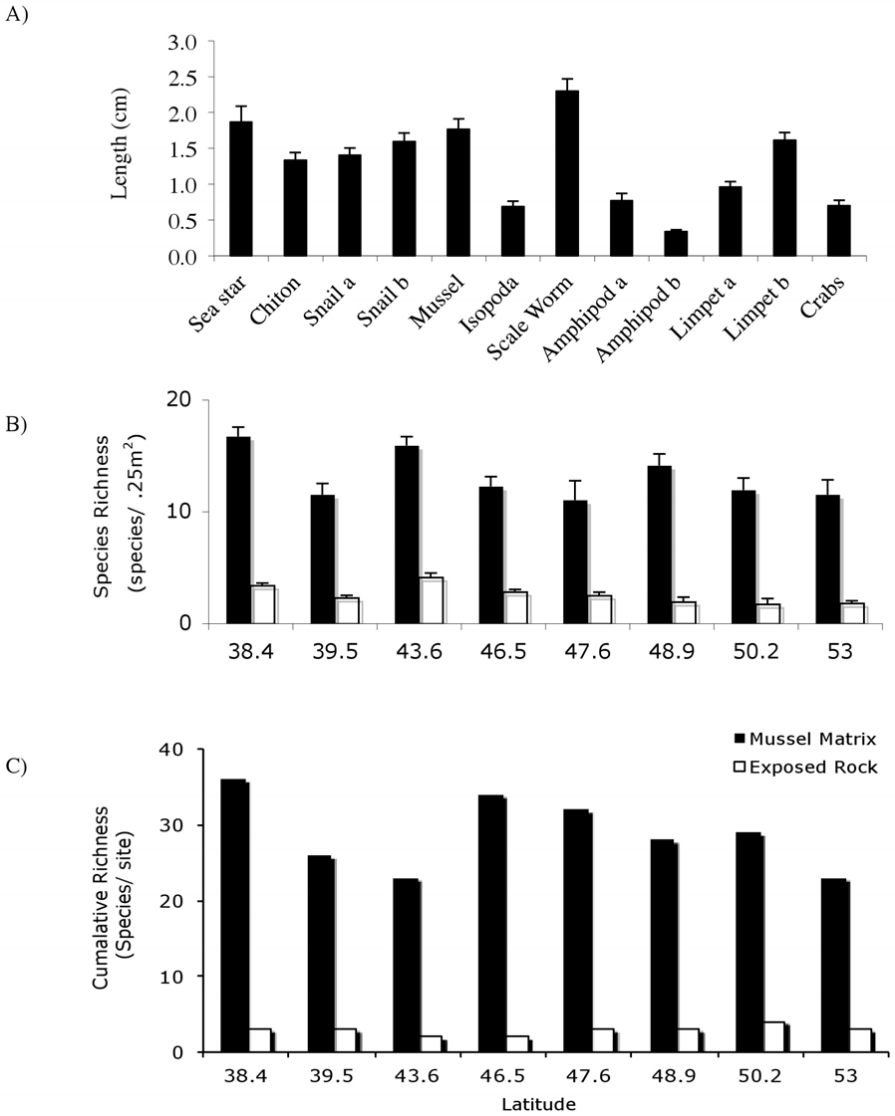
Diversity and structure of the intertidal community dependent on mussel matrix for shelter from harsh climate. (A) Average body length of the most common invertebrates found living inside or embedded next to (only limpets) mussel beds. Scientific names of species measured, with x-axis labels in parentheses, were: *Anasterias minuta* (sea star), *Plaxiphora aurata* (chiton), *Pareuthria plumbea* (snail a), *Trophon geversianus* (snail b), juvenile *Perumytilus purpuratus* (mussel), *Exosphaeroma lanceolata* (isopod), multiple species including *Halosyndna Patagonica*, *Mapphysa aenea* and *Platynereis magalhaensis* (scale worm), species names of amphipod species *a* and *b* are unknown, *Siphonaria lessoni* (limpet a), *Nacella magellanica* (limpet b), *Cyrtograpsus altimanus* and *Halicarcinus platinus* (crab);. (B) Species richness of invertebrates found living inside mussel matrix and on bare surfaces at 8 different sites spanning 2100 km of Argentinean coastline, from Northern Patagonia to Rio de Gallegos, Tierra del Fuego. (C) Cumulative species richness at those same sites. Bars are +/−1 SE.

In summer 2002, we explored the rocky shorelines of Cabo Dos Bahias. From the high to low intertidal on the exposed headlands, >95% of the rocky shore's surface was covered by one species, the tiny (∼2 cm) mussel, *Perumytilus purpuratus* (Fig. 1; [25]). The diverse intertidal life and zonation characteristic of most exposed rocky shore systems throughout the world were noticeably missing, as were large, mobile invertebrate predators. However, embedded and living within the 2–3 mussel-thick, rock-covering bio-matrix, we found a diverse assemblage complete with diminutive representatives (0.5–3 cm in length; see Fig. 2A) of the most common rocky shore animal groups (see above). Upon exposure to the intense Patagonian winds, crabs, polycheates and chitons laid on nearby bare rock quickly died, while the body mass of seastars receded noticeably. Based on these field observations, we hypothesized that: (1) the thick mussel matrix covering the exposed rocky shores of Patagonia, Argentina protects associated animals from lethal, wind-driven desiccation stress and (2) maintenance of biodiversity of rocky shore invertebrates depends exclusively on facilitation by foundation species, rather than keystone predation, under intense climate stress.

### Generality Surveys

To test the generality of our initial observations at Cabo Dos Bahias that rocky shore invertebrate communities are dependent on living inside the mussel matrix for persistence, we surveyed 8 rocky coast sites from southern to northern Patagonia, spanning over 2100 km in range. Our initial site was near the city of Rio de Gallegos adjoining to the Straights of Magellan in southern Patagonia. Our most northern site was ∼200 km north of Viedma, a city in northern Patagonia. Exact latitudes are given in Fig. 2, and each represented an unshaded, basaltic rock or sandstone outcropping. At each site, we randomly placed and then surveyed 10, 50×50 cm quadrats in both mussel and non-mussel occupied areas at or near mean low water. For non-mussel covered areas (which were uncommon), we recorded all species present, the densities of those species and the average size of the first five individuals of each species collected. For mussel-covered areas, the mussel matrix was carefully excavated and dissected, and all contents put into dishpans and sorted to determine all species present, their densities, and average size (i.e., mean length of first five individuals).

### Survivorship experiments

To test: (1) the hypothesis that mussels facilitate animal persistence in the Patagonian rocky intertidal and (2) the relative role of desiccation vs. consumer protection provided by mussels in this facilitative process (although we did not find observational evidence that mussels protected matrix associates from predation, we tested this mechanism as well because it is very common in other mussel-dominated systems), we performed controlled experiments at both exposed headland sites and one additional site ∼1 km away in the late spring of 2003. The experiment consisted of six treatments (n = 15 replicates of each treatment): 1) mussel matrix removal (bare areas), 2) mussel matrix removal+consumer exclusion (bare areas+cage), 3) mussel matrix removal+cage control (bare areas+cage controls), 4) mussel matrix removal+consumer exclusion+mussel matrix (bare areas+cage full of mussels), 5) mussel matrix removal+consumer exclusion+mussel sun-block mimic (bare areas+cage with shade), and 6) mussel matrix removal+consumer exclusion+mussel sun-and-wind-block mimic (bare areas+cage with mussel-sized rocks or sponge). Bare areas had no cage structures. Total consumer removal (i.e., caged) plots were covered with a stainless-steel mesh (5 mm), consumer exclusion cage (15×15×4 cm, LxWxH) bolted to the substrate with a stainless-steel, centered bolt. Cage control plots were covered with similar cages, but with two sides removed to give consumers access. Shaded plots had cages with two layers of 0.62 cm^2^ Vexar mesh strapped to the top. For bare areas+cage with moist sponge treatments, the matrix-mimicking sponge was the same size as the cage and, before being placed in the cage, it was soaked in seawater and then squeezed drip-dry to mimic climate buffering by mussels. For bare areas+cage full of mussel treatments, cages were packed full of excavated mussels and then fastened to the rock. Live organisms in all plots were initially scraped from the surface with paint scrapers. For this study, we used the most abundant organisms in the system amenable to transplanting: limpets, snails, chitons, scale worms, seastars, anemones (attached to mussel shells) and crabs. Because all organisms moved <6 cm after being placed on the rock surface in a preliminary study (30 minutes), we did not use line and glue to attach animals to the surface and thus avoided associated artifacts with line tethering. Instead, we simply placed each organism in its assigned treatment free of artificial attachment. Experimental organisms were removed from a nearby mussel matrix∼one hour before the study, immediately placed in seawater, and kept immersed until being placed into assigned treatments. The study ran for the entire length of diurnal low tide in each zone (∼3 hours in the low intertidal and 5 hours in the high intertidal). After set-up (∼30 minutes), we left the sites completely to allow for any bird predation that may occur as both gulls and oyster catchers occasionally visit these sites (∼1 seagull/500 m of exposed shoreline and 1 pair of oyster catchers at each exposed headland site, Silliman et al., *pers. obs.*). However, before leaving the sites and before the experiment could be fully set-up, all crabs, seastars, and anemones appeared extremely desiccated and dead (e.g., crabs could no longer move their legs after ∼10 minutes and their dorsal carapace had sunk in). At the end of the experiment, we recorded whether organisms were alive or dead. Finally, we recorded change in drip-free, wet weight of polycheates, sea stars, and crabs over the length of the study using a battery-powered scale in the field. For analysis, we used survivorship (% of animals surviving in the 15 replicates at each of the three sites) as one data point value for that site and then compared mean survivorship across treatments at the three sites. Thus, for this experiment, n = 3.

### Disturbance-Recovery Experiments

To test: (1) the hypothesis that mussel beds facilitate rocky shore community development and (2) the relative role of desiccation vs. predation protection provided by mussels in this facilitative process, we performed disturbance-recovery experiments at both exposed headland sites in late spring of 2002. The experiment consisted of six treatments (n = 8 per treatment): 1) mussel matrix removal/disturbance (bare areas), 2) mussel matrix removal/disturbance+consumer exclusion (bare areas+cage), 3) mussel matrix removal/disturbance+cage control (bare areas+cage controls), 4) mussel matrix removal/disturbance+consumer exclusion+mussels (bare areas+cage full of mussels), 5) mussel matrix removal/disturbance+consumer exclusion+mussel sun-block mimic (bare areas+cage with shade), and 6) mussel matrix removal/disturbance+consumer exclusion+mussel sun-and-wind-block mimic (bare areas+cage with mussel-sized rocks). Mussel-sized rocks were collected from a nearby field. Before use, mussels were thoroughly rinsed in freshwater and only mussels within 0.2 cm of the mean width and length of adult mussels where used in the caging experiment as mussel mimics. Rocks were packed tight enough inside cages so that there was no movement of rocks when waves crashed over top of the cages, which mimicked conditions in mussel treatments. Bare areas had no cage structures. Total consumer removal plots were covered with a stainless-steel mesh (5 mm), consumer-exclusion cage (15×15×4 cm, LxWxH) bolted to the substrate with a stainless-steel, centered bolt. Cage control plots were covered with similar cages, but with two sides removed to give predators access. Shaded plots had cages with two layers of 0.62 cm^2^ vexar mesh strapped to the top. Rocks used in the mussel-sized rock cage were taken from a nearby road. Cages with mussels were stocked with mussels that had been cleaned with seawater in dishpans and cleaned of all associated invertebrates. Live organisms in all plots were initially scraped from the surface with metal paint-scrapers and rock surfaces cleaned with a blow-torch. All plots were established in November of 2002, and the experiment ran for two years. Cages were checked and maintained monthly for limpet invasion and fouling as previously described. At the end of the experiment, we recorded species richness in each plot.

To test how seastars impact diversity of invertebrates within the mussel matrix, we performed a 1.5 yr predator removal experiment at both exposed sites at the interface between the low and mid intertidal zones where seastar abundance was highest. The experiment consisted of two treatments (n = 8 per treatment): 1) control mussels (cage+mussels) and 2) seastar removal (cage+mussels−seastars). At each site, 16–20×20 cm mussel plots were excavated, and all seastars were removed from the excavated mussels. All other invertebrates were retained. Excavated mussels with associated assemblages were then put into cages (as described above). In half of the 16 cages, 4 seastars were added to the mussel matrix to mimic natural densities. The 16 cages full of mussels and associated communities were then carefully fastened to the rock as described above so that no animals escaped. Sea star densities were check every 2 months. On average, 0.42+/−0.12 small seastars (<1 cm in length) were removed from exclusion plots, and 0.56+/−0.39 small seastars from inclusion plots to maintain treatment densities. The experiment ran for 1.5 years and, at the end of the experiment, all caged plots were excavated and species richness and densities were recorded.

### Desiccation assays

To test how mussels mitigate desiccation stress on Patagonian rocky shores and to parse out the relative importance of the effects of sun block and sun+wind block by mussels in reducing desiccation stress, we performed a sponge-evaporation study at the interface between the mid and low intertidal zones at two exposed headland sites in the Cabo Dos Bahias preserve. The headlands are separated by ∼1 km, and we conducted the experiment in both the spring and fall of 2003. The experiment consisted of five randomly assigned treatments (n = 8/treatment): 1) bare areas (controls), 2) bare areas+cage with shade (sun block only), 3) bare areas+cage (as a cage control), 4) bare areas+cage with moist sponge (sun block+wind block), and 5) bare areas+cage full of mussels. Bare control areas had no cage structures. Caged plots were covered with a stainless steel mesh (5 mm) cage (15×15×4 cm, LxWxH) bolted to the substrate with a stainless-steel, centered bolt. Shaded plots had cages with two layers of 0.62 cm^2^ vexar mesh strapped to the cage top. For bare areas+cage with moist sponge treatments, the matrix-mimicking sponge was the same size as the cage and, before being placed in the cage, it was soaked in seawater and then squeezed drip-dry to mimic climate buffering by mussels. For bare areas+cage full of mussel treatments, cages were packed full of excavated mussels and then fastened to the rock. Live organisms in all plots were initially removed from the surface with metal paint-scrapers and wire brushes. On the rock surface in each treatment at the beginning of the study, we placed a numbered, wet, 5×10×2 cm sponge. The dry and wet weight of each sponge was determined before the experiment. The study ran for 2.5 h on a clear day with <10% cloud cover and with an average wind speed of 43.2 km/h+/−5.4 km. After 2.5 h, each sponge was collected and immediately weighed in the field with a battery-powered scale. Percent water loss was determined by: [(initial wet weight−final wet weight)/(initial wet weight−dry weight)]*100.

### Statistical analyses

Latitudinal survey data (body length and richness) were analyzed with a two-way ANOVA (latitude×substrate type) or with chi square analyses (cumulative richness - latitude×substrate type) and desiccation data with a two-way ANOVA (season×cage type). Data from tethering experiments were analyzed using a two-way ANOVA (tidal elevation×cage type). Caging data from the disturbance recovery experiment were analyzed using a three-way (site×zone×cage type) and from the seastar predation experiment using a two-way ANOVA (predator presence×site). In analyses, data either exhibited homogeneity of variance and were normally distributed or were transformed using log transformations for assumption conformity. Only linear contrasts were compared, using Tukey's post hoc test. Because we found no significant effect of site (P>0.26 all cases) on any response variable in all experiments, data were pooled across all sites for all experiments.

## Results

### Generality Surveys

**Figure 3:**
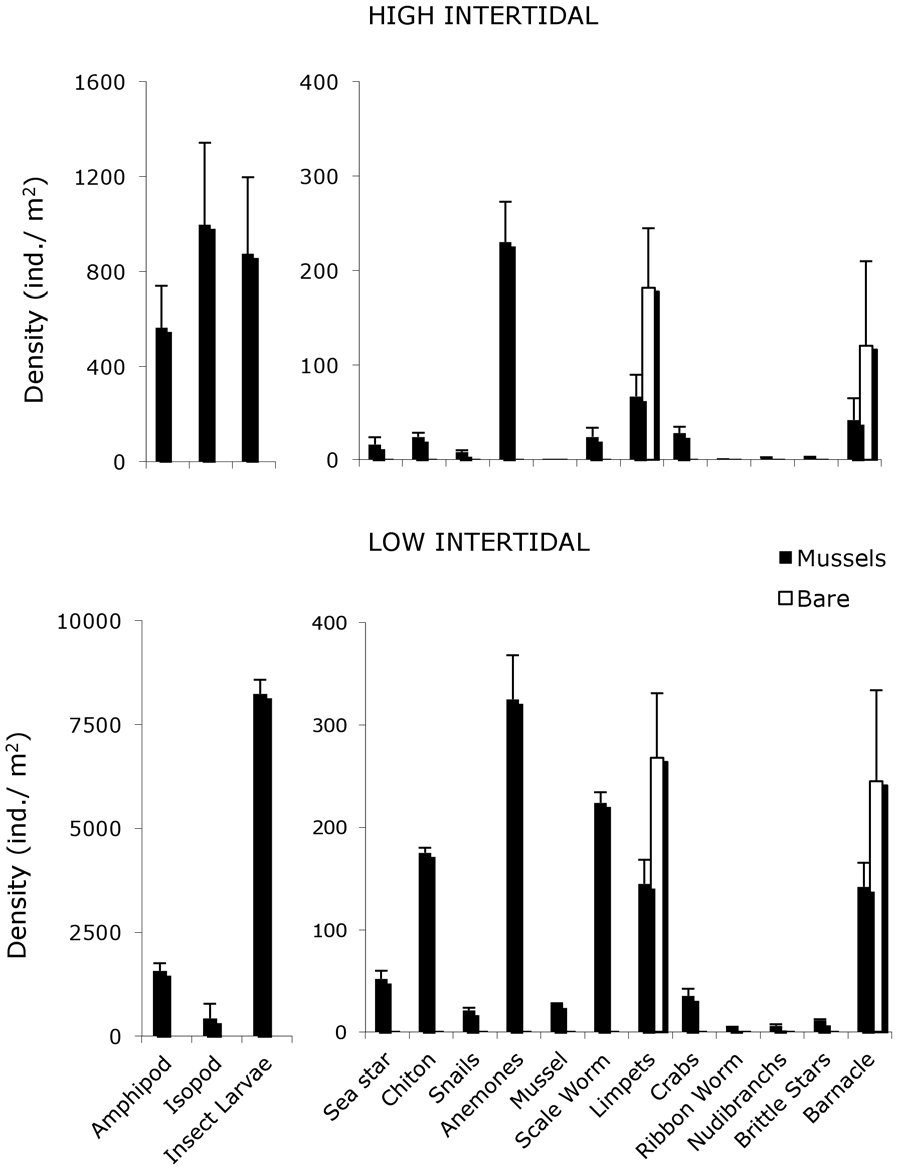
Density of invertebrates found living inside mussel matrix at the main experimental sites. Densities of amphipods, snails and limpets all consisted of aggregates of 2 species (i.e., *a* and *b*); scientific names of taxa not found in Fig. 2 are *Oulactis muscosa*, *Aulactinia sp.* and *Phymactis sp.* (anemone), *Mytilus edulis* (mussel) Nemertea (ribbon worm), Nudibranchia (nudibranchs), Ophiuiridea (brittle star), *Balanus glandula* and *B. laevis* (barnacle). All other x-axis labels correspond directly to species names listed in Fig. 2 legend. Bars are +/−1 SE.

Analysis of our species richness survey data revealed that there was no interaction between factors (mussel presence×latitude; P>0.35) or impact of latitude (P>0.17), only a significant effect of mussel presence (P<0.01). At each site, on average, 28.9 species were found within mussel beds while only 2.7 species were found on rock surfaces free of mussels (Fig. 2). The number of species per quadrat was nearly 6× higher within mussel beds (Fig. 2). Species abundance differences between bare rock and mussel-covered areas were even more dramatic than species richness (Fig. 3). As was the case for species richness, for species abundances, there was no interaction between factors (P>0.42) or impact of latitude (P>0.19), only a significant effect of mussel presence (P<0.01). Species densities within mussel beds ranged from 2–7640 ind./m^2^, while on the bare surfaces they ranged from 0–262ind./m^2^ (invasive barnacles primarily, and some limpets). Typical rocky shore consumers that forage on bare rock and overtop mussel beds such as seastars, chitons, crabs, and shell-drilling snails were only found within the mussel matrix (Fig. 2, 3), while only 2 limpet species (which spend most of their time on the shore nestled into the edge of the mussel matrix and forage on open rock space during high tides), and an invasive barnacle, occurred in significant numbers outside of mussel beds (Fig. 3). The associational reliance of local species richness and abundance on mussel beds was consistent across all intertidal heights (Figs. 2, 3). Body size measurements revealed that all organisms were less than 2.5 cm in mean body length and the dominant adult seastar and crab were on average 1.83 and 0.84 cm long, respectively (Fig. 2A).

### Survivorship Assays

**Figure 4:**
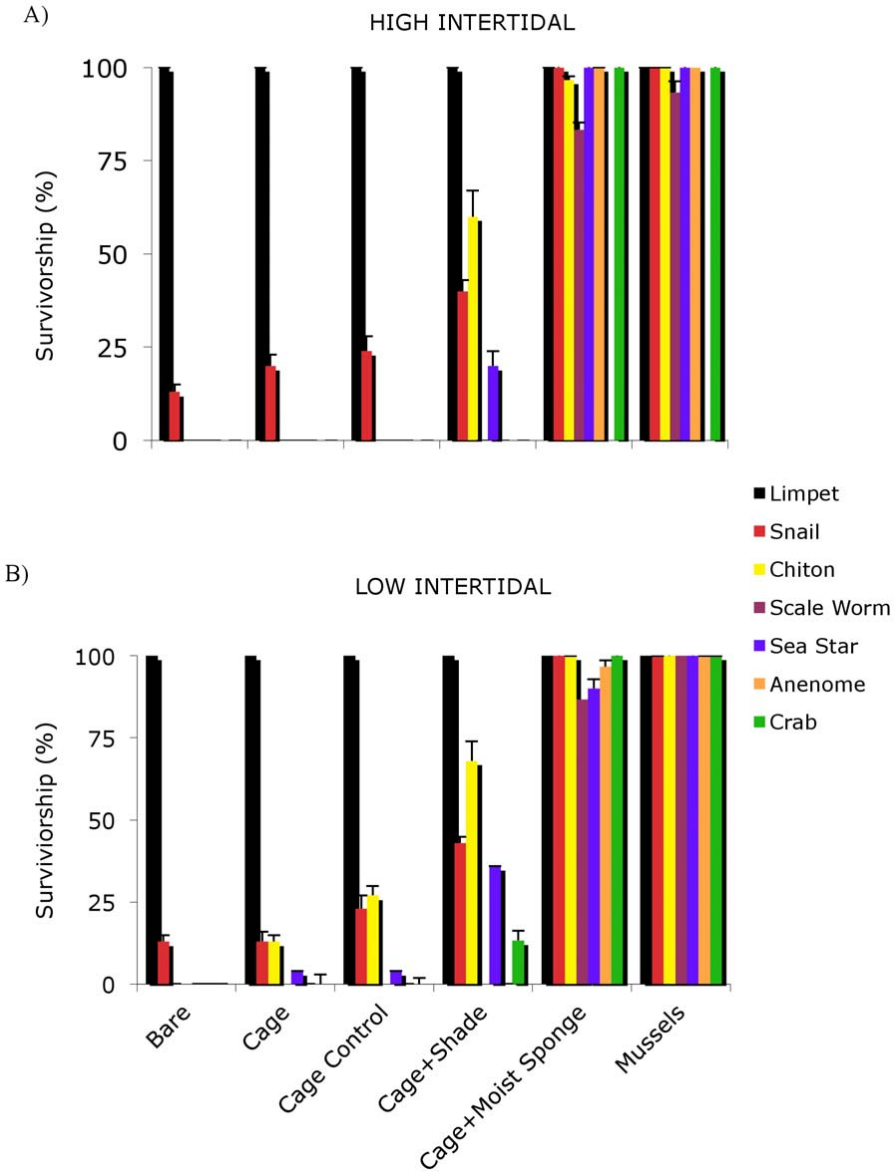
Survivorship assays demonstrating the dependence of marine invertebrates on facilitation by mussels. Effects of mussel presence and mimics of the positive effects of mussel presence (predator and desiccation refuge) on survivorship of the most common invertebrates living in the mussel matrix on Patagonian exposed shorelines in both the (A) high and (B) low intertidal. Invertebrate taxa consisted of single or multiple species. Bars are +/−1 SE.

In our survivorship experiments, for limpets, there was no effect of tidal elevation or caging treatment on survivorship (P>0.33) and survivorship was 100% across all treatments (Fig. 4). For chitons, there were significant main effects (P<0.05, both cases) of caging and tidal elevation, with chitons surviving better at low elevations and under cages with added shade, mussels, or sponges. For scale worms, snails, sea stars, anemones and crabs there was only a significant effect of caging treatment (P<0.01, all cases; Fig. 4), with survivorship being higher under cages with added shade, mussels, or sponges. Importantly, cages with shade increased survivorship for all animals (except limpets) only moderately, from 0 to ∼10–40%, while adding either mussels or a moist sponge to cages placed on top of tethered organisms elevated survivorship to nearly 100% for all non-limpet organisms (Fig. 4). For drip-free weight changes, polycheates, sea stars, and crabs all lost greater than 60% of their body weight after 1.5 hours (P<0.01).

### Disturbance-recovery and Predation Experiments

**Figure 5:**
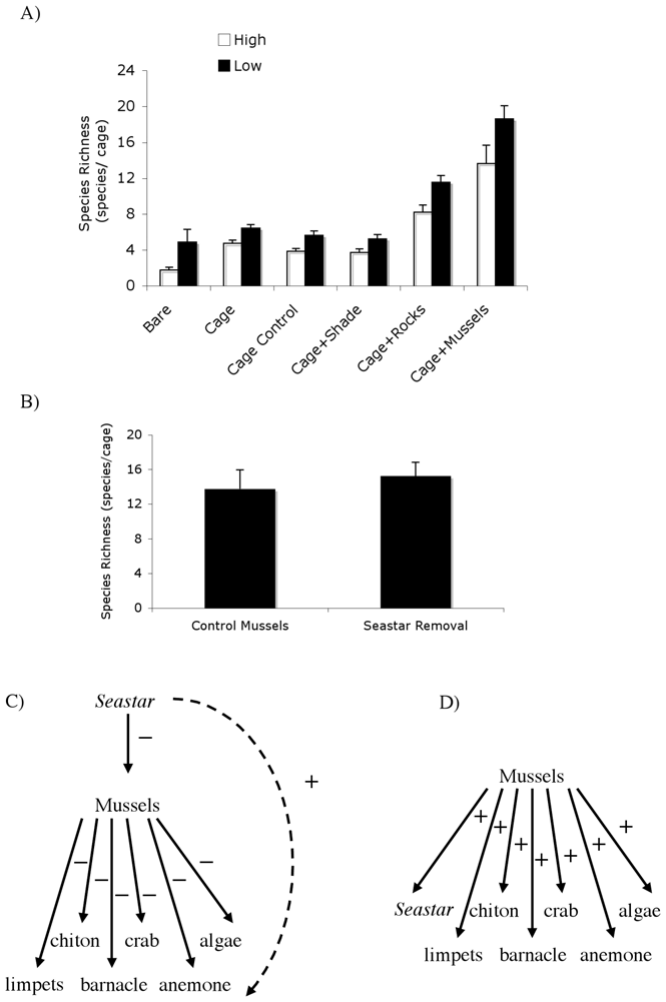
Importance of facilitation by mussels for the resilience and persistence of Patagonian intertidal rocky shore community. (A) Effects of mussel presence and mimics of the positive effects of mussel presence (i.e., refuge from predators and desiccation) on recovery of invertebrate community after experimental disturbance. (B) Effect of seastar removal on diversity of interstitial invertebrates in mussel beds. (C) Schematic of trophic cascade network that regulates diversity on moist, rocky shores throughout the world. (D) Schematic of shift in biodiversity maintenance to a facilitation network under a regime of intense climatic stress. Bars are +/−1 SE.

In our 2-year disturbance-recovery experiment, there was a significant effect of both cage type (P<0.001) and zone (P<0.05) on species richness, but no interaction (Fig. 5A). Species richness increased with increasing protection from climatic extremes, while there was no positive effect of removing consumers on richness that could not be explained by the positive effects of physical-stress buffering afforded by cage structure (cage vs. cage controls). Cage+shading treatments provided no positive effect on species richness, and it was only when cages were filled with three-dimensional objects (mussels and rocks) did the community significantly recover from disturbance. Across all treatments, being located at lower elevations increased species richness by ∼25%. After 1.5 years, there was no effect of seastar removal (P>0.48) on species richness inside the mussel matrix (Fig. 5B).

### Desiccation stress test

For the evaporation potential experiment, there was a significant effect of cage type (P<0.001) and no effect of season (P>0.46). Loss of water from small wet sponges placed in the intertidal was intense (>90%) in plots with no protection from wind or sun. By contrast, desiccation stress was dramatically reduced within the mussel matrix, as well as in the mussel bed mimic of a large wet sponge. In shading plots, water loss was reduced by only <10%, revealing that primary loss of water was driven by wind, not sun stress.

## Discussion

**Figure 6:**
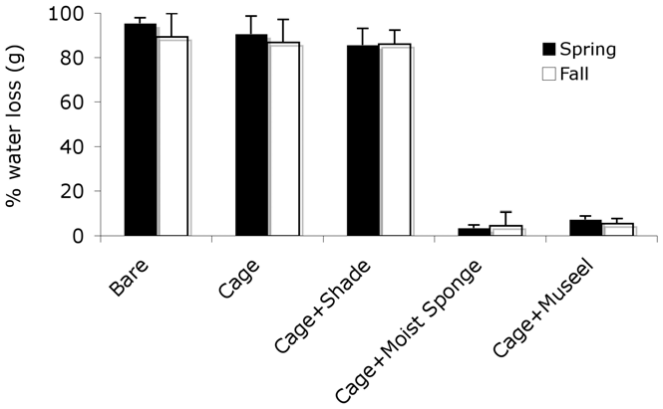
Facilitative effects of mussel and mussel mimics on reducing desiccation stress. Bars are +/−1 SE.

Our surveys across 15° of latitude of wind-swept Patagonia rocky shorelines revealed a striking example of whole-community facilitation. Diversity was 20-fold higher in plots where mussels were present; in the absence of mussels, the rocky substrate was virtually bare. This whole-community, mussel-bed diversity relationship occurred across the entire intertidal zone and did not diminish across the decreasing evaporative stress gradient from high to low intertidal that characterizes wave-exposed, rocky shore systems (i.e., decreasing desiccation stress at lower elevations). Our survey also revealed that the dominant mussel and its invertebrate associates are extremely diminutive in body size (Fig. 2 - all organisms were less than 2.5 cm in mean body length), an order of magnitude smaller than other rocky shore assemblages with similar taxa [31]. Concomitant with reduced body size and the observed positive mussel bed – species diversity and abundance association (Fig. 2, 3), desiccation stress in this system is the highest ever recorded for any rocky intertidal system [25]. Experimental manipulation of sun and wind exposure in both high and low intertidal habitats during spring and fall of 2003 combined with mathematical estimates of desiccation stress from daily local weather data over a 3-year period [25] revealed that the primary force driving evaporative water loss in this system was exposure to low-humidity, high-intensity winds (>90% of evaporation due to winds), not sun (Fig. 6).

Experimental examination of the positive mussel bed–local diversity association confirmed our hypotheses that mussels facilitate the entire local community by buffering against harsh, lethal climatic conditions. Survival assays revealed that ambient desiccation stress was quickly lethal to native intertidal animals and that diversity was dependent on mussel facilitation (Fig. 4). Local crabs, seastars, chitons, scale worms, and anemones all died within 5–180 minutes of air exposure on rock surfaces without mussels. Our results show that excluding consumers had no impact on the survivorship of organisms, as there was no difference between caged and open areas that could not be explained by the small, positive effects of caging generated by slight habitat amelioration provided by cage controls. In contrast, when intertidal animals were covered with mussels, or moist sponges, survivorship was nearly 100% for all species, at both high and low elevations, demonstrating that water loss is the primary mechanism leading to death of exposed invertebrates.

Like persistence, community resilience (ability to recover from disturbance) was completely dependent on facilitation by mussels (Fig. 5A). After two years, no significant recovery took place in bare plots without mussels. Only the few species commonly found outside mussel beds (e.g. barnacles and limpets) were found in bare areas, huddled on mussel matrix edges outside of the disturbance plot. By contrast, in mussel areas cleared of interstitial organisms, diversity after two years of recovery was 6× times higher, with 17.8 species per plot. Cumulatively, across disturbance-recovery plots, 5 species were found in bare areas, whereas 36 were found in mussel treatments. Removing all consumers had no impact on community recovery (Fig. 5A), except for the small benefits that cage-edges provided in full cages and cage controls in reducing desiccation stress [25]. Shading also had no impact on community recovery (Fig. 5A). Mussel-mimicking rocks, however, increased species richness by 4×, nearly the same positive effect generated by the mussel bed. This contrasting result of no impact of shading but strong, positive impacts of both mussel beds and mussel-mimics combined with the results from evaporation-potential experiments (Fig. 6) and calculations [25] demonstrates that crevice space among mussels facilitates community development by decreasing lethal desiccation stress generated by the dry, intense, Patagonian winds.

Experimental removal of diminutive predatory seastars from the mussel matrix also had no detectable effect on species richness (Fig. 5B), confirming that under the intensified climatic stress at our study sites predation is not playing an important diversity-regulating role. This result, together with a recent study on these same shorelines showing that the Patagonian mussels experience almost no predation over the entire year [32], reveals that predation pressure on mussels is weak in this intertidal system. Indeed, the only native, marine predators found to forage in the intertidal, and then only during high tides, were tiny decapod crabs (1–2 cm in body length) and ntothenid fish (∼9 cm in body length); examination of these animals' gut contents revealed amphipods and polycheates, and not mussels, were their primary source of nutrition. Our observation of the absence of mobile consumers from this coastline could have multiple and potentially interacting causes. Intense physical stress could prevent many rocky shore predators (e.g., large seastars) from emerging on the shorelines or prevent their establishment after recruitment. Alternatively, intertidal predators could be absent from this system due to historical contingencies (L Orensanz and M. Adami, unpub. manus.). Finally, large, sub-tidal mammalian (sea lions, porpoises), bird (penguins), molluscan (octopus) and fish predators in the area could suppress densities of intertidal predators, such as crabs and sea stars. Despite finding no experimental evidence for strong predator effects in our study system and no observational evidence for the presence of effective intertidal predators over large spatial scales in our surveys, we caution against a large-scale generalization that predation is functionally absence on Patagonian shorelines. Indeed, this hypothesis needs to be tested experimentally across many sites before such broad conclusions can be made. In other intertidal Argentinian communities, for instance, consumers have been shown to be important over large spatial scales [33], [34].

Mussel beds on wave-exposed rocky shores worldwide have been shown to have both suppressive and facilitative effects on local diversity. For example, in the absence of keystone predation by sea stars mussel beds competitively displace other common intertidal space-holders such as barnacles, anemones, and algae by preempting space, which leads to negative impacts of mussels on the diversity of other dominant space-holding species [13]. By providing habitat and protection from desiccation, mussel beds also facilitate organisms like worms, echinoderms, and cnidarians, extending their distributions higher into the intertidal zone by reducing desiccation stress [35]. On wind-swept Patagonian shorelines, our experiments and large-scale survey reveal that competitive exclusion of other species by mussels becomes irrelevant because mussels are the *only* dominant space holder capable of enduring the physical conditions; indeed, virtually all other intertidal organisms are completely dependent on mussels for protection from lethal desiccation stress for their persistence (even limpets cannot survive the drying winds without access to mussel matrix edges over extended periods) [36]. Furthermore, predation pressure at our two study sites is weak even in the presence of buffered conditions provided by mussels. Our results therefore agree with predictions of environmental stress models [21], [22], [37] and suggest that with increasingly severe climatic conditions, local diversity maintenance tends towards whole-community facilitation while the role of predation is diminished.

Mutualistic and facilitative interactions that form networks (3 or more interacting species) are key for generating and maintaining patterns of biodiversity [38], [39], and consumers have the potential to dictate the relative importance of these positive interaction networks for the maintenance of diversity [40]. Our experimental work reveals the persistence of a diverse community on exposed, arid Patagonian shorelines despite the functional absence of keystone predation. Our findings provide experimental evidence for theoretical predictions that basal species in ecological networks can still persist when not regulated by predation, competition, or resource availability [41], as the diverse inhabitants of mussel beds (chitons, anemones, amphipods etc.) on exposed, Patagonian shorelines are instead controlled by harsh climactic conditions and the positive interaction network that ameliorates those conditions. Physical factors have been shown to dampen the strength of keystone predation [21], [42]. Our study expands this knowledge to show that climactic extremes can not only suppress keystone predation entirely, but also foster the dominant role of facilitation in community organization and diversity maintenance (Fig. 5C, D).

Over the past decade, ecologists have recognized that foundation species amelioration of physical stress maintains local diversity in many natural communities that are subject to continuing human impact (e.g., salt marshes, coral reefs, forests) [43]. Failure to appreciate the increasingly important role that foundation species will play in maintaining local biodiversity as climatic stress intensifies could have dire consequences for the persistence and resilience of natural ecosystems. Recent climatic models predict a doubling of the most recent, best-estimates of global temperature increases over the next 100 years [44]. This steep and rapid temperature rise is expected to increase evaporation and wind stress in many natural and manmade ecosystems around the globe, including economically important shoreline communities, such as dunes, mangroves, and marshes and inland communities, such as grasslands, farmlands and savannahs. Our results suggest that conservation efforts in these areas where increased climate stress is expected should incorporate and promote positive species interactions, especially whole-community facilitation by foundation species, that can buffer biodiversity from harsh physical conditions.

## References

[1] Vitousek PM, Mooney HA, Lubchenco J, Melillo JM (1997). Human domination of Earth's ecosystems.. Science.

[2] MEA (2005). Millennium Ecosystem Assessment. Ecosystems and human well-being: Current state and trends.

[3] Byrnes JE, Reynolds PL, Stachowicz JJ (2007). Invasions and Extinctions Reshape Coastal Marine Food Webs.. PLoS ONE.

[4] Naeem S, Bunker DE, Hector A, Loreau M, Perrings C (2009). Biodiversity, Ecosystem Functioning, and Human Wellbeing. An Ecological and Economic Perspective.

[5] Chapin FS, Zavaleta ES, Eviner VT, Naylor RL, Vitousek PM (2000). Consequences of changing biodiversity.. Nature.

[6] Silliman BR, van de Koppel J, Bertness MD, Stanton L, Mendelsohn I (2005). Drought, snails, and large-scale die-off of southern U.S. salt marshes.. Science.

[7] Worm B, Barbier EB, Beaumont N, Duffy JE, Folke C, Halpern BS (2006). Impacts of biodiversity loss on ocean ecosystem services.. Science.

[8] Barbier EB, Koch EW, Silliman BR, Hacker SD, Wolanski E (2008). Coastal ecosystem-based management with nonlinear ecological functions and values.. Science.

[9] Klanderud K, Totland O (2005). Simulated climate change altered dominance hierarchies and diversity of an alpine biodiversity hotspot.. Ecology.

[10] McClain CR, Barry JP (2010). Habitat heterogeneity, biogenic disturbance, and resource availability work in concert to regulate biodiversity in deep submarine canyons.. Ecology.

[11] Tylianakis JM, Didham RK, Bascompte J, Wardle DA (2008). Global change and species interactions in terrestrial ecosystems.. Ecology Letters.

[12] Kiers ET, Palmer TM, Ives AR, Bruno JF, Bronstein JL (2010). Mutualims in a changing world: an evolutionary perspective.. Ecology Letters.

[13] Paine RT (1966). Food web complexity and species diversity.. American Naturalist.

[14] Connell JH (1978). Diversity in tropical rain forests and coral reefs.. Science.

[15] Sousa WP (1979). Disturbance in marine intertidal boulder fields: the nonequilibrium maintenance of species diversity.. Ecology.

[16] Bruno JF, Stachowicz JJ, Bertness MD (2003). Inclusion of facilitation into ecological theory.. Trends in Ecology & Evolution.

[17] Dayton PK, Parker BC (1972). Toward an understanding of community resilience and the potential effects of enrichments to the benthos at McMurdo Sound.. Proceedings of the colloquium on conservation problems in Antarctica.

[18] Stachowicz JJ (2001). Mutualism, facilitation, and the structure of ecological communities.. Bio Science.

[19] Angelini C, Altieri AH, Silliman BR, Bertness MD (in press). Interactions among foundation species underlie community organization.. Bio Science.

[20] Altieri AH, Silliman BR, Bertness MD (2007). Hierarchical organization via a facilitation cascade in intertidal cordgrass bed communities.. American Naturalist.

[21] Menge BA, Sutherland JP (1987). Community regulation: variation in disturbance, competition, and predation in relation to environmental stress and recruitment.. American Naturalist.

[22] Bertness MD, Callaway R (1994). Positive interactions in communities.. Trends in Ecology & Evolution.

[23] Power ME, Tilman D, Estes JA, Menge BA, Bond WJ (1996). Challenges in the quest for keystones.. Bio Science.

[24] Hart DD (1992). Community organization in streams: the importance of species interactions, physical factors, and chance.. Oecologia.

[25] Bertness MD, Crain CM, Silliman BR, Bazterrica MC, Reyna MV (2006). The community structure of western Atlantic Patagonian rocky shores.. Ecological Monographs.

[26] Seed R (1969). The ecology of Mytilus edulis L. (Lamellibranchiata) on exposed rocky shores. II. Growth and mortality.. Oecologia.

[27] Paine RT (1971). A short-term experimental investigation of resource partitioning in a New Zealand rocky intertidal habitat.. Ecology.

[28] Lubchenco J, Menge BA (1978). Community development and persistence in a low rocky intertidal zone.. Ecological Monographs.

[29] Paine RT, Castillo JC, Cancino J (1985). Perturbation and recovery patterns of starfish-dominated intertidal assemblages in Chile, New Zealand, and Washington State American.. Naturalist.

[30] Bruno JF (2000). Facilitation of cobble beach plant communities through habitat modification by Spartina alterniflora.. Ecology.

[31] Denny MW, Gaines S (2007). Encyclopedia of Tidepools and Rocky Shores.

[32] Hidalgo FJ, Silliman BR, Bazterrica MC, Bertness MD (2007). Predation on the rocky shores of Patagonia, Argentina.. Estuaries and Coasts.

[33] Silliman B, Bortolus A (2003). Underestimation of Spartina productivity in western Atlantic marshes: marsh invertebrates eat more than just detritus.. Oikos.

[34] Alberti J, Escapa M, Daleo P, Iribarne O, Silliman BR (2007). Local and geographic variation in grazing intensity by herbivorous crabs in SW Atlantic salt marshes.. Marine Ecology Progress Series.

[35] Suchanek TH, Moore PG, Seed R (1986). Mussels and their role in structuring rocky shore communities.. The Ecology of Rocky Coasts.

[36] Bazterrica MC, Silliman BR, Hidalgo FJ, Crain CM, Bertness MD (2007). Limpet grazing on a physically stressful Patagonian rocky shore.. Journal of Experimental Marine Biology and Ecology.

[37] Fortuna MA, Bascompte J (2006). Habitat loss and the structure of plant–animal mutualistic networks.. Ecology Letters.

[38] Bascompte J, Jordano P, Olesen JM (2006). Asymmetric coevolutionary networks facilitate biodiversity maintenance.. Science.

[39] Verdu M, Valiente-Banuet A (2008). The Nested Assembly of Plant Facilitation Networks Prevents Species Extinctions.. American Naturalist.

[40] Palmer TM, Stanton ML, Young TP, Goheen JR, Pringle RM (2008). Breakdown of an ant-plant mutualism follows the loss of large herbivores from an African Savanna.. Science.

[41] Brose U, Berlow EL, Martinez ND (2005). Scaling up keystone effects from simple to complex ecological networks.. Ecology Letters.

[42] Sanford E (1999). Regulation of keystone predation by small changes in ocean temperature.. Science.

[43] Halpern BS, Silliman BR, Olden JD, Bruno JP, Bertness MD (2007). Incorporating positive interactions in aquatic restoration and conservation.. Frontiers In Ecology and the Environment.

[44] Sokolov AP, Stone PH, Forest CE, Prinn R, Sarofim MC (2009). Probabilistic forecast for 21st century climate based on uncertainties in emissions (without policy) and climate parameters.. Journal of Climate.

